# Supplementary material for: Antibody activities in hyperimmune plasma against the Rhodococcus equi virulence -associated protein A or poly-N-acetyl glucosamine are associated with protection of foals against rhodococcal pneumonia
Source: PLoS One. 2021 Aug 26;16(8):e0250133. doi: 10.1371/journal.pone.0250133 (PMC8389416; doi:10.1371/journal.pone.0250133)
Supplement: Attachment — Submitted filename: Response Letter PLoS One June 14_2021.pdf [file pone.0250133.s002.pdf]

COLLEGE OF VETERINARY MEDICINE
& BIOMEDICAL SCIENCES

Department of Large Animal Clinical Sciences
4475 TAMU

TEXAS A&M UNIVERSITY

June 12, 2021

Prof. Ashlesh K. Murthy
Academic Editor
PLoS One

Subject: Revision of manuscript entitled, "**Antibody Activities in Hyperimmune Plasma Against the *Rhodococcus equi* Virulence -Associated Protein A or Poly-*N*-Acetyl Glucosamine are Associated with Protection of Foals Against Rhodococcal Pneumonia**"

Dear Dr. Murthy:

Thank you for your electronic message dated June 11, 2021 about the above-referenced manuscript. I also thank the reviewers who carefully considered our report. My coauthors and I have revised the report on the basis of the reviewer's comments that were provided to us. Below, we detail how each point raised by the reviewer was addressed in the revised report. The revised report with marked changes and an unmarked manuscript will be uploaded to the *PLoS One* website along with this response letter.

Comments from Reviewer 1:

1. Do these farms historically use plasma as a preventative measure?

AUTHORS' RESPONSE: We thank the reviewer for pointing out this important omission. The manuscript has been revised to indicate that both farms historically used *R. equi* hyperimmune plasma transfusion to control *R. equi* pneumonia.

2. Who was responsible for allocating foals to the treatment groups? Was allocation sequence concealed from the person assigning foal to treatment groups? Were any demographics on the foals recorded?

AUTHORS' RESPONSE: We thank the reviewer for raising more great points. Allocation of treatment was made by investigators at Texas A&M University. In the fall of 2018, each farm provided a list of the eligible mares at the farm ordered by expected breeding date. These mares were randomly assigned in a balanced manner to either plasma 0 or 1. The randomly assigned list was provided to the veterinarians at the farm. Because the true foaling date did not always match the expected foaling date, the order of treatment was further randomized by nature. We arrived at this system based on internal discussions with investigators and statisticians and farm personnel. The manuscript has been revised to clarify who was responsible for the treatment allocation and that those administering plasma were aware of which foal was assigned to which plasma.

We have also revised the report to indicate which additional data (including sex of the Quarter Horse foals) were collected for each foal.

3. Who was responsible for thoracic ultrasound examinations? Who administered the plasma? Were any complications noted following administration of either type of plasma?

AUTHORS' RESPONSE: The manuscript has been revised to indicate that thoracic ultrasonography was performed by the farm veterinarians (Drs. Blodgett, Canaday, and Turner). The manuscript also has been revised to indicate that plasma transfusion was performed by veterinary technical staff or veterinarians at the farm. The manuscript has been revised to indicate that no adverse effects were noted among transfused foals. We thank the reviewer for prompting us to address each of these important points.

4. What was the standard treatment protocol for subclinical/clinical cases?

AUTHORS' RESPONSE: The standard antimicrobial treatment at both farms was a macrolide with or without combination with rifampin. The manuscript has been revised to include these details for each farm.

5. Line -234 how was data that was obtained from this foal before it was lost handled? Was it excluded from the analysis?

AUTHORS' RESPONSE: We apologize for the ambiguity. The manuscript has been revised to clarify that the data from this foal were excluded from analysis.

6. Line 284 – does the percentage of foals that developed pneumonia reflect those that developed clinical and subclinical pneumonia? Please clarify in the main text and the figure legend.

AUTHORS' RESPONSE: We apologize for the ambiguity. The manuscript has been revised to clarify that the percentage of foals includes those with clinical pneumonia (Farm A) and subclinical pneumonia (Farm B).

7. Figure 4: assuming each dot represents an individual animal for Farm B, I count 10 data points for this that developed pneumonia, whereas the text says 9. Please clarify.

AUTHORS' RESPONSE: We apologize for the confusion. The extra point was actually an artifact from the same symbol being used to indicate an outlier as was used to reflect individual foals (*i.e.*, an extra dot was added to indicate that foal's value was an outlier). The figure was revised to correct this problem.

8. Figures 7 and 8, please consider labeling the Y-axis.

AUTHORS' RESPONSE: The Y axis has been labelled in revised Figures 7 and 8.

9. Did all the foals that developed clinical or subclinical survive? Was there any difference in survival for those treated with PNAG HIP vs. RE HIP?

AUTHORS' RESPONSE: We have revised the manuscript to indicate that no study foals died of pneumonia caused by *R. equi*. The third paragraph from the end of the Discussion indicates that there was no significant difference in protection between the 2 plasma products; however, these results should be interpreted with caution because the study was not designed to test this hypothesis.

10. Can you please describe the roles of the authors affiliated with Mg Biologics and Alopexx Inc. in the study design, excitation, analysis, and manuscript preparation? Was the plasma provided as gift from these companies?

AUTHORS' RESPONSE: Thank you for these comments. The editor also has asked that this be addressed in the Funding Statement and Competing Interest Statements. Neither Mg Biologics nor Alopexx played a role in the design, execution, analysis, or manuscript preparation for this study EXCEPT that Mg Biologics agreed to make labels masking the plasma identity and to not break the code for the plasma until data collection was completed. Vaccine to hyperimmunize donors against PNAG was provided as a gift by ALOPEXX, Inc. The plasma was purchased by the farms from Mg Biologics.

Again, we thank you and the reviewer for taking the time to carefully consider our report. Please let us know if you have any additional questions or concerns regarding the revised manuscript.

Sincerely,

Noah D. Cohen, VMD, MPH, PhD, Dipl. ACVIM-LA
Professor and Associate Department Head for Research and Graduate Studies
